# Quality of Care for Maternal and Newborn Health in Health Facilities in Nepal

**DOI:** 10.1007/s10995-019-02846-w

**Published:** 2019-12-17

**Authors:** Ashish KC, Dipendra Raman Singh, Madan Kumar Upadhyaya, Shyam Sundar Budhathoki, Abhishek Gurung, Mats Målqvist

**Affiliations:** 1 International Maternal and Child Health, Department of Women’s and Children’s Health, Uppsala University, Uppsala, Sweden; 2 Ministry of Health, Government of Nepal, Kathmandu, Nepal; 3 School of Public Health and Community Medicine, B.P. Koirala Institute of Health Sciences, Dharan, Nepal; 4 Golden Community, Lalitpur, Nepal

**Keywords:** Nepal, Maternal and neonatal survival, Quality of care, Service readiness and availability

## Abstract

**Introduction:**

Nepal has pledged to substantially reduce maternal and newborn death by 2030. Improving quality of intrapartum health services will be vital to reduce these deaths. This paper examines quality of delivery and newborn services in health facilities of Nepal.

**Methods:**

Data were sourced from the Nepal Health Facility Survey 2015, which covered a national representative sample of health facilities. The datasets were analysed to assess service readiness, availability and quality of delivery and newborn care in a sample of 992 health facilities.

**Results:**

Of the 992 facilities in the sample, 623 provided delivery and newborn care services. Of the 623 facilities offering delivery and newborn care services, 13.3% offered comprehensive emergency obstetric care (CEmONC), 19.6% provided basic emergency obstetric care (BEmONC) and 53.9% provided basic delivery and newborn service. The availability of essential equipment for delivery and newborn care was more than 80% in health facilities. Except for the coverage of vitamin K injection, the coverage of immediate newborn care was more than 85% in all health facilities. The coverage of use of chlorhexidine ointment to all newborns was more than 70% in government hospitals and primary health care centers (PHCCs) and only 32.3% in private hospitals.

**Conclusions:**

These findings show gaps in equipment and drugs, especially in PHCCs and private health facilities. Improving readiness and availability of equipment and drugs in PHCCs and private health facility will help improve the quality of care to further reduce maternal and newborn mortality in Nepal.

## Significance

The global Every Newborn Action Plan (ENAP) lays out a plan to improve quality of care (QoC) at and around the time of birth that is critical for further reducing maternal and neonatal mortality. This paper explores the current state of the quality of maternal and neonatal care in Nepal’s health facilities.

## Introduction

Two global strategic plans, the ENAP and Strategies for Ending Preventable Maternal Mortality (EPMM), aim to reduce preventable maternal and newborn deaths and stillbirths by 2030 across the Sustainable Development Goal (SDG) period (World Health Organization and UNICEF 2014; World Health Organization 2015). This can only be realized by improving QoC during pregnancy, intrapartum and postpartum periods (United Nations 2015, 2016; Boerma et al. 2018). During the Millennium Development Goal (MDG) period, annual coverage estimates of maternal and newborn health (MNH) services from MDG priority countries (as reported in the *Countdown to 2015* report) provided impetus through increased accountability for investments (UNICEF 2015; Victora et al. 2016). As a result, 40 million more births now take place in health facilities than in 2001 (Boerma et al. 2018). In 2017, globally, 80% of pregnant women had access to at least one antenatal care (ANC) service while 60% had access to skilled delivery at birth and 35% had access to postnatal care (PNC; Boerma et al. 2018). These improvements made a large contribution to the 29% reduction in maternal mortality from 390,185 women in 2000 to 275,288 in 2015 (Kassebaum et al. 2016). The neonatal mortality rate reduced by 42% in the same period (Wang et al. 2016). Further reductions in maternal mortality require improved QoC for MNH (Kruk et al. 2016; Persson 2017).

The Institute of Medicine (IOM) defines QoC as “the degree to which health services for individuals and populations increase the likelihood of desired health outcomes and are consistent with current professional knowledge” (Institute of Medicine Committee on Quality of Health Care in America 2001). The Lancet Global Health Commission defined high-quality health system is “one that optimises health care in a given context by consistently delivering care that improves or maintains health outcomes, by being valued and trusted by all people, and by responding to changing population needs” (Kruk et al. 2018).

Improving QoC in a health facility is a two-step process. First, the facility needs to be adequately ready to provide care through infra-structure, equipment, drugs and human resource. Second, provision of the high-quality care requires implementation of set of quality improvement interventions (Andriantsimietry et al. 2016, Kanyangarara et al. 2017; Ashish et al. 2019).

Over the past two decades, the Government of Nepal has improved access to MNH care by expanding community-based health services. Efforts have also been made to reduce financial barriers to access care, including the introduction of free health services and establishment of National Health Insurance. In many cases, however, the expansion of health services has not been accompanied by improved quality at the point of care. Health care providers in Nepal face significant challenges in providing adequate care, including understaffing and a lack of equipment and supplies. Managers of health facilities are poorly prepared and inadequately supported to fulfil their roles successfully (Ministry of Health, Nepal 2017).

At the strategic and policy level, the issue of “quality at the point of service delivery” has gained enormous visibility through the emphasis placed on universal coverage of basic health services in the Constitution of Nepal 2015. QoC is one of the four strategic principles of the Nepal Health Sector Strategy 2015–2020 (NHSS; Ministry of Health, Nepal 2016). The strategy identified the three needed outputs of (i) setting care standards, (ii) ensuring internal quality improvement mechanisms for implementing standards of care, and (iii) establishing an independent quality assurance system to assess QoC periodically (Ministry of Health, Nepal 2016).

Understanding the state of service readiness, availability and QoC during delivery and birth will provide evidence for designing quality improvement interventions to improve QoC in Nepal. The Ministry of Health and Population in 2015 conducted a national level survey, NHFS to assess the service readiness, availability and QoC in health facilities of Nepal to generate evidence for better programming. This paper is a secondary analysis to the NHFS to examine health system readiness, availability and quality of maternal and newborn services in Nepal’s health facilities.

## Methods

The study carried out the secondary analysis of data from the nationally representative NHFS 2015. The NHFS was a cross-sectional study that was representative of the main types of health facilities in Nepal. The 2015 NHFS was based on generic designs and modules developed by the Measure DHS, ICF Macro. The core tools were revised and modified to the country context and aligned with WHO’s Service Availability and Readiness Assessment (SARA) and the Ministry of Health’s Service Tracking Survey (STS).

### Data Collection

The NHFS is representative for different facility types [public hospitals, primary health care centres (PHCCs), health posts, urban health centres (UHCs), HIV testing and counselling sites (HTC), and private hospitals], for different managing authorities (government and private), and for each of Nepal’s six administrative regions and three ecological regions [mountain, hills and plains (terai)]. For the primary data collection, Ministry of Health and Population selected 992 health facilities from the master list of 4719 facilities. The master list included 101 government hospitals, 364 private hospitals, 204 PHCCs, 3771 health posts and 279 non-government organization (NGO)-run and other health facilities.

Due to the non-proportional allocation of the sampled health facilities to the different domains and to the different health facility types, sampling weights were required for the analysis to ensure the actual representation of the study results. Sampling weights were calculated separately based on sampling probabilities for each sampling stratum. The health facility design weight was adjusted for non-response at the sampling stratum level to obtain the health facility sampling weight. The sampling weight was then normalized at the national level to calculate the health facility standard weight. The normalization of the sampling weight is intended to ensure that the total number of unweighted cases equals the total number of weighted cases at the national level (Ministry of Health, Nepal 2016).

Assessment of the facilities providing delivery and newborn care service was done through direct observation by independent data collectors. Direct observation was performed to determine the availability of equipment and drugs. The equipment assessed was delivery bed, partogram, thermometer, foetal stethoscope or pinard, adult stethoscope, digital blood pressure apparatus, manual blood pressure apparatus, bag and mask (neonatal) and baby weighing scales. The drugs used during labour and delivery were assessed using an observation checklist. The drugs assessed were injectable antibiotics (ceftriaxone, ampicillin), injectable uterotonics, vitamin K injection, tetracycline eye ointment for newborns and chlorhexidine ointment for umbilical cleansing. The quality of newborn services was assessed using an observation checklist. The observation checklist was pretested for reliability. Based on the consistency and validity of the tool for use, the observation checklist was used to collect data in this study.

### Data Management

From the NHFS primary dataset, we extracted following domain for the study purpose:

- Number of health facilities which provided delivery and newborn care service and categorizing by type of health facility, managing authority, province and ecological region.
  Immediate newborn care-skin to skin contact immediate after birth in mother’s abdomen, drying and wrapping of baby, initiation of breast feeding within first hour of birth, weighing of newborn after birth, essential newborn care before discharge, administration of injectable vitamin K, application of chlorhexidine ointment to umbilical stump, application of tetracycline ointment to eyes, BCG vaccination; routine examination (head to toe) of newborns.

### Data Analysis

We analysed the availability of intrapartum and newborn services by

- *Health facility type* government hospitals, private hospitals, PHCCs and health post;
- *Managing authority* government owned, NGO, private for profit and faith-based organization;
- Location of hospital by province 1 to 7;
- *Ecological region* mountain, hill and terai;
- *Health facility providing delivery and newborn service* Comprehensive Emergency Obstetric and Neonatal Care (CEmONC), Basic Emergency Obstetric and Neonatal Care (BEmONC) and Birthing centres.

Descriptive tables were generated from the NHFS dataset on the availability of intrapartum and newborn services on intrapartum and newborn by type of facility, management authority province and ecological area. The availability of equipment to provide delivery services by type of facility was also analysed. We assessed the performance of health workers on immediate and essential newborn care before discharge. We assessed availability of the partogram (pictorial record) and neonatal resuscitation kit by type of facility.

## Results

Among the 992 health facilities which were surveyed, 62.8% (623) provided delivery and newborn service. Among the type of health facilities, only 49.9% of health posts provided the delivery and newborn services. Among the seven provinces, province 2 had lowest proportion (50.8%) of health facilities providing delivery and newborn care services (Table 1).

**Table 1:** Health facilities that provide delivery and newborn service

Variable	Types of variable	Providing delivery and newborn care	
		N	Weighted %
Facility type	Government hospitals	76/79	94.1
	Private hospitals	96/166	64.3
	Primary health care centres (PHCCs)	192/200	95.3
	Health post	235/471	43.6
Managing authority	Government and public	525/771	47.4
	NGO and private not-for-profit	19/70	22.6
	Private for profit	75/118	62.3
	Mission and faith-based	4/4	100.0
Provinces	1	117/178	47.0
	2	67/132	23.0
	3	136/232	41.4
	4	63/88	56.3
	5	90/146	44.8
	6	63/80	82.9
	7	87/126	73.6
Earthquake-affected	No	495/739	49.9
	Yes	128/224	47.6
Ecological region	Mountain	93/135	56.8
	Hill	314/435	56.1
	Terai	216/371	32.6
Total		623/992	

Of the 623 facilities offering delivery and newborn care services, 13.4% offered comprehensive emergency obstetric care (CEmONC), 19.6% provided only basic emergency obstetric care (BEmONC) and 53.9% provided basic delivery and newborn services as birthing center (Table 2).

**Table 2:** Health facilities providing delivery and newborn services

Province	CEmONC n (weighted %)	BEmONC n (weighted %)	Birthing center n (weighted %)	Other^a^ n (weighted %)	No. of facilities providing delivery and newborn care
1	18 (15.4)	22 (18.8)	64 (54.7)	13 (11.1)	117
2	8 (11.9)	11 (16.4)	38 (56.7)	10 (14.9)	67
3	21 (15.4)	21 (15.4)	61 (44.8)	33 (24.3)	136
4	8 (12.7)	19 (30.2)	32 (50.8)	4 (6.3)	63
5	12 (13.3)	22 (24.4)	49 (54.4)	7 (7.8)	90
6	7 (11.1)	13 (20.6)	38 (60.3)	5 (7.9)	63
7	10 (11.5)	14 (16.1)	54 (62.1)	9 (10.4)	87
Total	84 (13.4)	122 (19.6)	336 (53.9)	81 (13.0)	623

*CEmONC* comprehensive emergency obstetric care, *BEmONC* basic emergency obstetric care

^a^Facilities that did not provide delivery and newborn services

The availability of equipment for delivery and newborn care in the health facilities varied by type of health facility. Among the government hospitals providing delivery and newborn services, more than 90% of them had delivery bed, blank partogram, thermometer, fetal stethoscope or pinard, blood pressure apparatus, and baby weighing machine and 80% of them had bag-and-mask devices for neonatal resuscitation. Among the private health facilities providing delivery and newborn service, 84.8% of them had a blood pressure apparatus, 79.7% of them had stethoscopes and 81.2% of them had foetal stethoscope or pinard. The availability of essential drugs for mother and newborn care varied by the type of health facilities. Among the government hospitals, all had injectable uterotonics, 83% had injectable antibiotics, 72.4% had chlorhexidine ointment and 19.7% had injectable injection vitamin K. Among the private hospitals, 87.5% had injectable uterotonics, 68.8% had injectable antibiotics, 29.2% had chlorhexidine ointment and 55.2% had vitamin K injection. Among the PHCCs, 90.6% had injectable uterotonics, 55.7% had injectable antibiotics, 61.5% had chlorhexidine ointment and 2.6% had vitamin K injection (Table 3).

**Table 3:** Availability of essential equipment and drugs in labour and delivery room

Items n (weighted %)	No. of facilities providing delivery and newborn care (n = 623)	Government hospitals (n = 76)	Private hospitals (n = 96)	Primary health care centres (n = 192)	Health posts (n = 235)
Equipment					
Delivery bed	607 (97.4)	76 (100.0)	92 (95.8)	189 (98.5)	226 (96.2)
Blank partogram	519 (83.3)	74 (97.4)	61 (63.5)	169 (88.0)	192 (81.7)
Thermometers	503 (79.6)	70 (92.1)	81 (84.4)	148 (77.1)	184 (78.3)
Foetal stethoscope or pinard	571 (80.7)	75 (98.7)	80 (83.3)	177 (92.2)	219 (93.2)
Stethoscope	559 (89.7)	75 (98.7)	84 (87.5)	167 (87.0)	210 (89.4)
Digital blood pressure apparatus	16 (2.6)	6 (7.9)	6 (6.2)	2 (1.0)	2 (0.9)
Manual blood pressure apparatus	535 (83.8)	75 (98.7)	83 (86.4)	158 (82.3)	16 (6.8)
Bag and mask (neonatal)	562 (90.2)	76 (79.8)	82 (85.4)	180 (93.8)	200 (85.1)
Baby weighing scales	585 (93.9)	76 (100.0)	87 (90.6)	185 (96.4)	213 (90.6)
Drugs					
Injectable antibiotic (e.g. ceftriaxone, ampicillin)	332 (53.3)	63 (82.9)	66 (68.8)	107 (55.7)	79 (33.6)
Injectable uterotonic (e.g. oxytocin)	563 (90.4)	76 (100.0)	84 (87.5)	174 (90.6)	205 (87.2)
Vitamin K injection	79 (12.7)	15 (19.7)	53 (55.2)	5 (2.6)	2 (0.9)
Tetracycline eye ointment for newborns	196 (31.5)	22 (28.9)	12 (12.5)	55 (28.7)	100 (42.6)
Chlorhexidine ointment (umbilical stump cleansing)	370 (59.4)	55 (72.4)	28 (29.2)	118 (61.5)	153 (65.1)

**Fig. 1:**
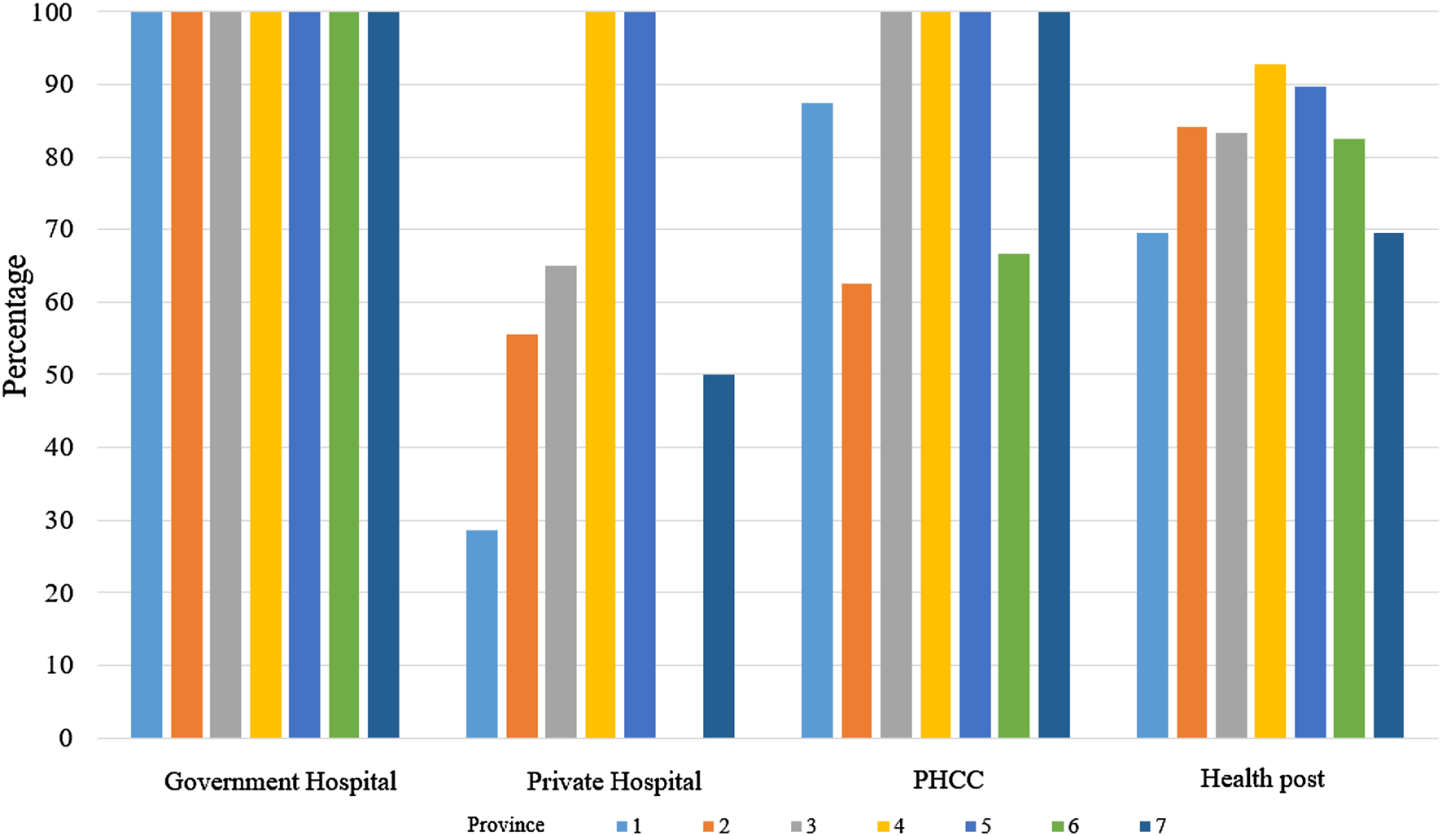
Availability of partogram for intrapartum care in labour rooms of health facilities by province

**Fig. 2:**
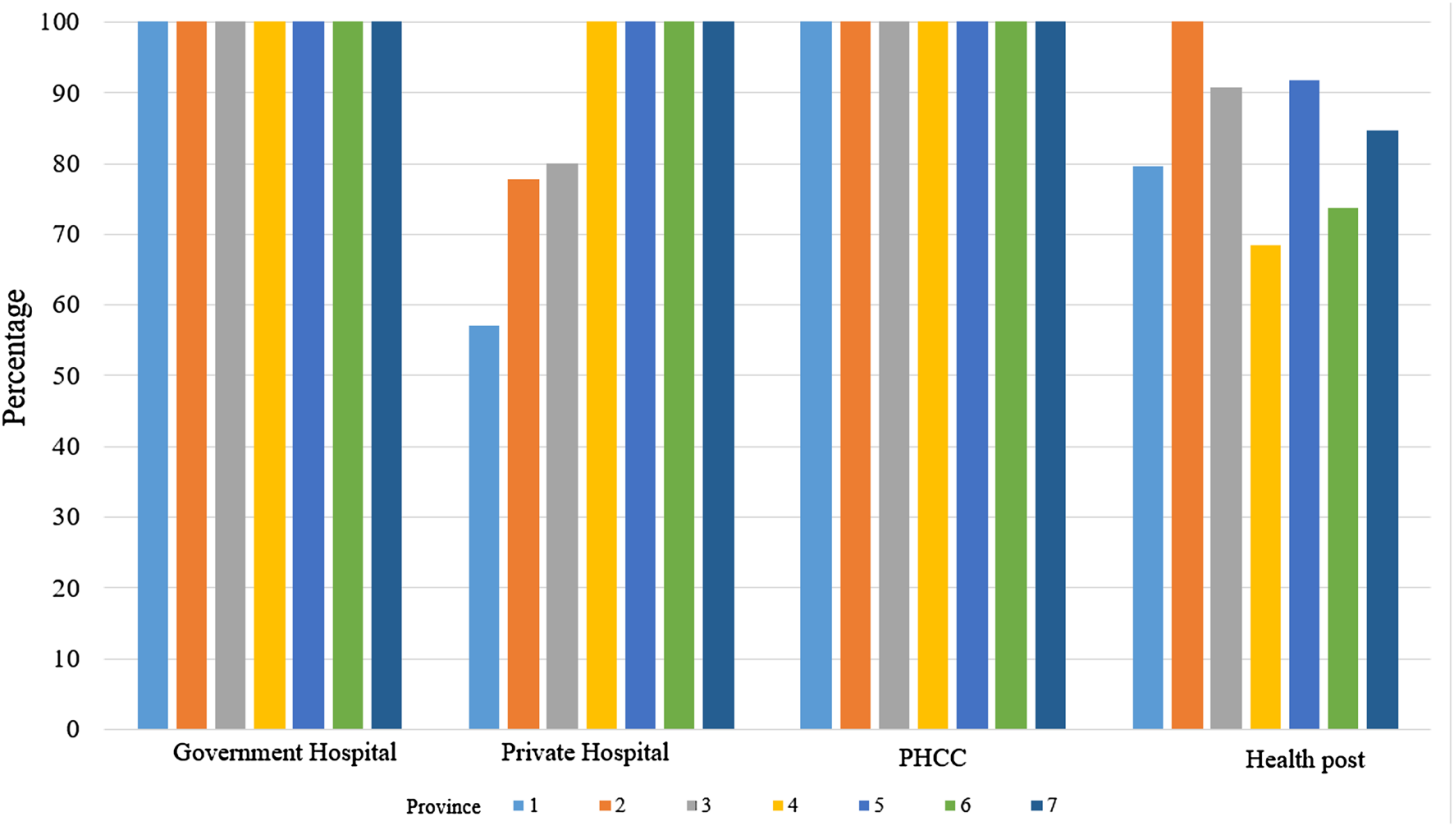
Availability of bag and mask (neonatal) neonatal resuscitation equipment in delivery room of health facilities by province

Neonatal resuscitation and partogram for intrapartum care were more available in higher level than lower level facilities (Figs. 1, 2). Coverage of newborn care immediately after birth and before discharge varied by type of health facilities. Except for the coverage of vitamin K injection, the coverage of immediate newborn care was more than 85% in all health facilities. Coverage of injection vitamin K to newborns was 4.7% in PHCC, 17.1% in government hospitals and 70.4% of private hospitals. The coverage of use of chlorhexidine ointment to all newborns was more than 70% in government hospitals and PHCCs and 32.3% in private hospitals (Table 4).

**Table 4:** Newborn care practices routinely performed at delivery facilities (NHFS 2015)

	All delivery providers n = 623 (%)	Government hospitals n = 76 (%)	Private hospitals n = 96 (%)	Primary health care centres n = 192 (%)	Health posts n = 235 (%)
Immediate newborn care					
Delivered to the abdomen (skin-to-skin)	572 (91.8)	68 (89.5)	86 (89.6)	181 (94.3)	217 (91.2)
Dried and wrapped newborns to keep warm	615 (92.9)	76 (100.0)	94 (97.9)	192 (100.0)	229 (96.9)
Initiated breastfeeding within first hour	618 (99.2)	75 (98.7)	94 (97.9)	192 (100.0)	233 (99.0)
Weighed newborns immediately upon delivery	608 (97.6)	76 (100.0)	94 (97.9)	191 (99.5)	223 (94.9)
Administration of vitamin K to newborns	104 (16.7)	13 (17.1)	69 (71.2)	9 (4.7)	6 (2.1)
Newborn care before discharge					
Applied chlorhexidine to umbilical stump	402 (64.5)	54 (71.1)	31 (32.3)	137 (71.4)	164 (69.8)
Applied tetracycline eye ointment to both eyes	78 (12.5)	11 (14.5)	6 (6.3)	25 (13.0)	32 (13.6)
Gave newborn BCG vaccine	102 (16.4)	26 (34.2)	23 (24.0)	22 (11.5)	25 (10.6)
Routine examination (head-to-toe) of newborns	604 (97.0)	75 (98.7)	92 (95.8)	191 (99.5)	222 (94.5)

## Discussion

The findings on service readiness, availability and QoC for mothers and newborns in health facilities provide impetus for improving QoC. One in 11 health facilities in Nepal provide CEmONC services and 1 in 9 health facilities provide basic emergency obstetric services (BEmONC). The MNH services were available in all the types of health facilities; however, the availability of essential equipment, tools and drugs varies by type of facility. There tend to be more equipment available for intrapartum care in higher level health than the lower level facilities. Ensuring the availability of basic tools such as partogram and neonatal resuscitation equipment at all levels is critical for the better monitoring of labour and preparedness for intervening when complications and problems arise. The coverage of chlorhexidine ointment application to all newborn is better in the public than in the private hospitals, indicating the need to more widely disseminate and institute the updated standards for care in the private sector.

A similar national level health facility survey conducted in Tanzania in 2014–2015, showed that hospitals had better readiness and availability of services than health centres for delivery. Those hospitals which had quality improvement systems for care delivery had better readiness to provide basic emergency and obstetric care (Bintabara et al. 2019). A study conducted in Malawi to assess the association of health facility readiness to the quality of newborn showed that health facilities which had better preparedness for delivery and newborn care with equipment and drugs provided better newborn care (Carvajal-Aguirre et al. 2017).

The introduction of free institutional delivery and demand side financing for institutional delivery in the last 15 years in Nepal led to a fourfold increase in the proportion of deliveries occurring in health institutions (Ministry of Health and Population Nepal, New ERA, and ICF International 2007; Ministry of Health and Population, and ICF International 2012; Ministry of Health, Nepal 2017; Raven et al. 2011). However, a disproportionate increase in delivery care in large public hospitals in comparison to peripheral health facilities compromised the QoC in both settings. In large public hospitals, the quality of antepartum, intrapartum and postpartum care has been compromised by over-crowding and poor readiness to support the influx of clients (Ministry of Health and Population, Nepal 2013). In peripheral health facilities, despite improved readiness to provide MNH services, the retention of complex skills, such as the resuscitation of patients, remains a challenge due to low client flow (Dickson et al. 2014). Improving the QoC in both settings requires a deeper understanding of the current care and context-specific bottlenecks to the provision of quality care. Assessing the availability, readiness and quality of health care services provides information for effective health systems management (Raven et al. 2011). The delivery of quality services requires readiness to deliver them in health facilities.

There are several steps to improve the readiness, availability and QoC for mothers and newborns. First is to understand the barriers to readiness, availability and QoC in the hospital through a process of bottleneck analysis. The bottleneck analysis provides guidance on the areas where bottleneck exist for improving the care. Based on findings, developing and implementing the quality improvement plan to overcome the barrier will improve the QoC (Ashish et al. 2019).

The current study had several limitations. First, the measurement of the performance of health workers was only on essential newborn care and not for intrapartum care such as the use of partogram or resuscitation. Second, it did not examine the engagement of facilities’ management structures on decision making for improving QoC. Finally, the study did not provide the user experience on the care provided by health facilities, which is an important dimension in the QoC.

This study provides evidence on the quality of maternal and newborn care for a cross-section of Nepal’s health facilities. Many lower level facilities lacked readiness for maternal and newborn care. The United Nation’s Global Every Women and Every Child strategy aims for improved maternal, newborn and child survival and for all children to thrive such that their full potential is realized resulting in economic benefits for the nation (United Nations 2016). Improved quality of maternal and newborn care is essential not only for the survival of newborns but also for enabling them and their mothers to have better quality lives by preventing antepartum and intrapartum complications. Developing a strategy that addresses the barriers and enabling facility managers to oversee its implementation is crucially important for sustaining improvements.

## Abbreviation

BEmONC: Basic Emergency Obstetric and Neonatal Care
CEmONC: Comprehensive Emergency Obstetric and neonatal Care
ENAP: Every Newborn Action Plan
EPMM: Strategies for ending preventable maternal mortality
MNH: Maternal and newborn health
QoC: Quality of care
NHFS: Nepal Health Facility Survey
PHCC: Primary health care centres
SARA: Service Availability and Readiness Assessment
SDG: Sustainable Development Goal
STS: Service Tracking Survey
WHO: World Health Organization
UHC: Urban health centres
HTC: HIV testing and counselling sites
